# Supplementary material for: Synthesis, Characterization and Anticancer Efficacy Evaluation of Benzoxanthone Compounds toward Gastric Cancer SGC-7901
Source: Molecules. 2022 Mar 18;27(6):1970. doi: 10.3390/molecules27061970 (PMC8949258; doi:10.3390/molecules27061970)

Supporting information

Figure S1a ^{1}H NMR for compound **3a**

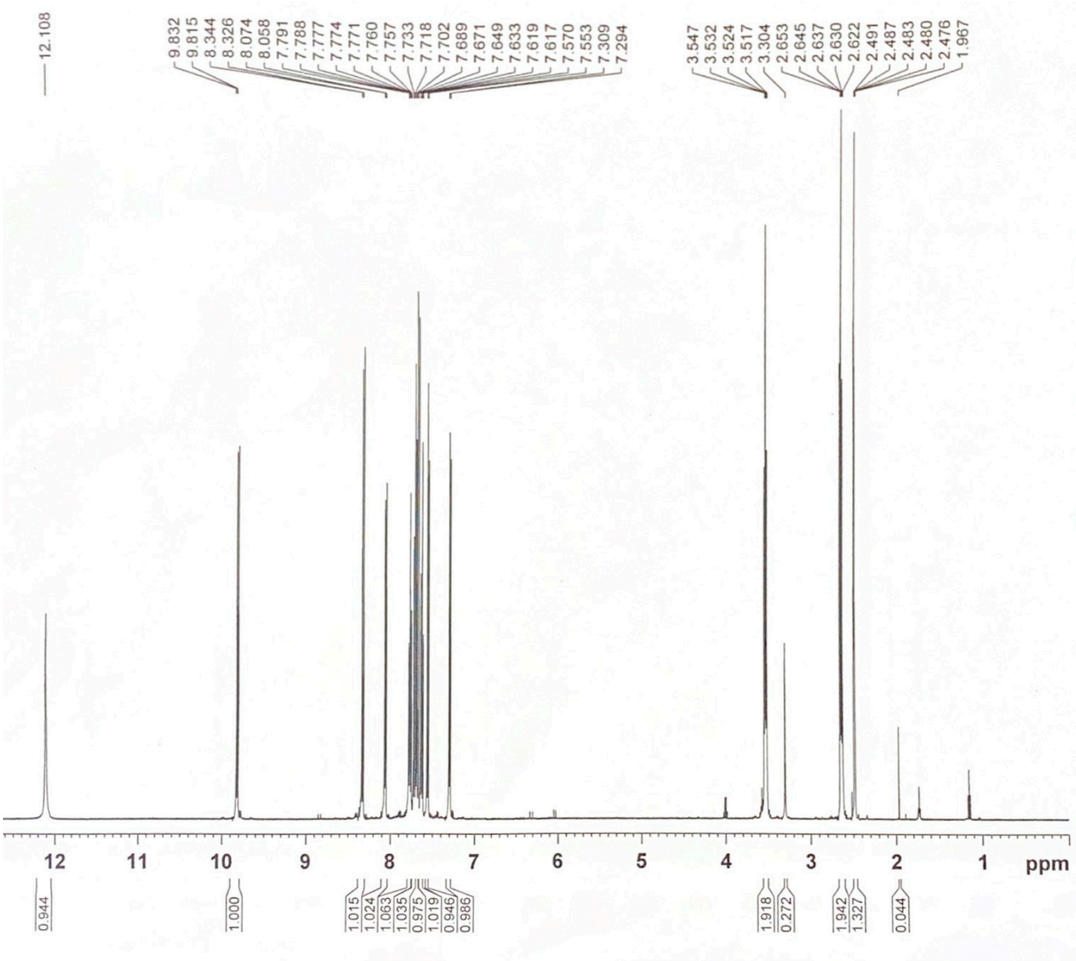

Figure S1b ^{13}C NMR for compound **3a**

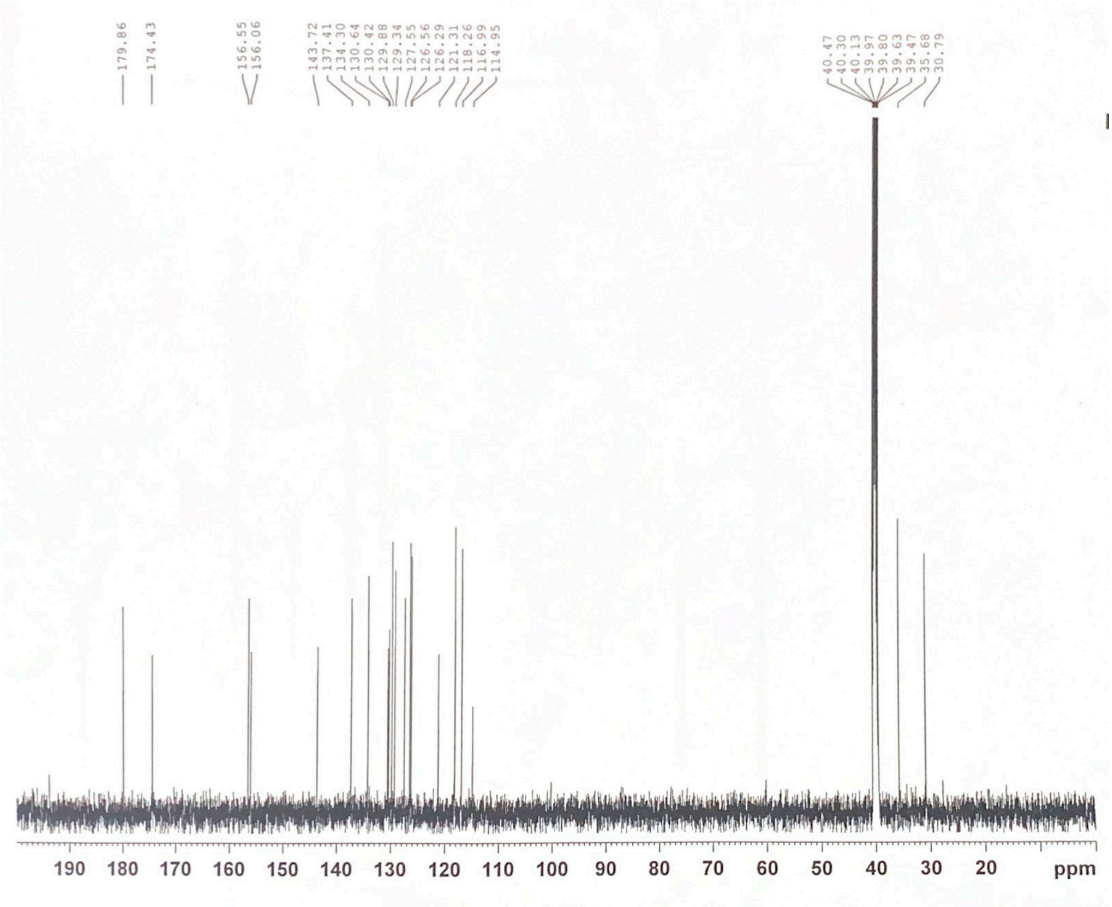

Figure S1c HRMS spectra for **3a**

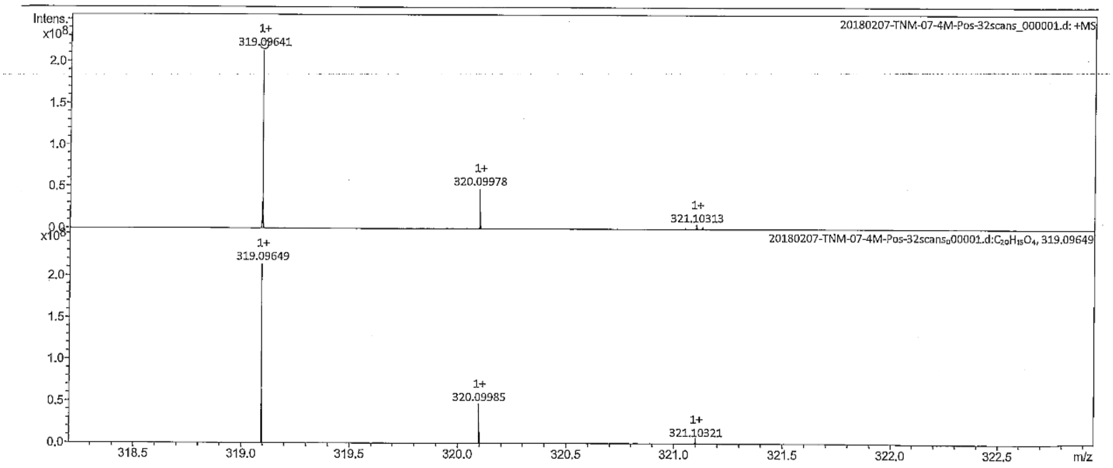

Figure S2a ^{1}H NMR for compound **3b**

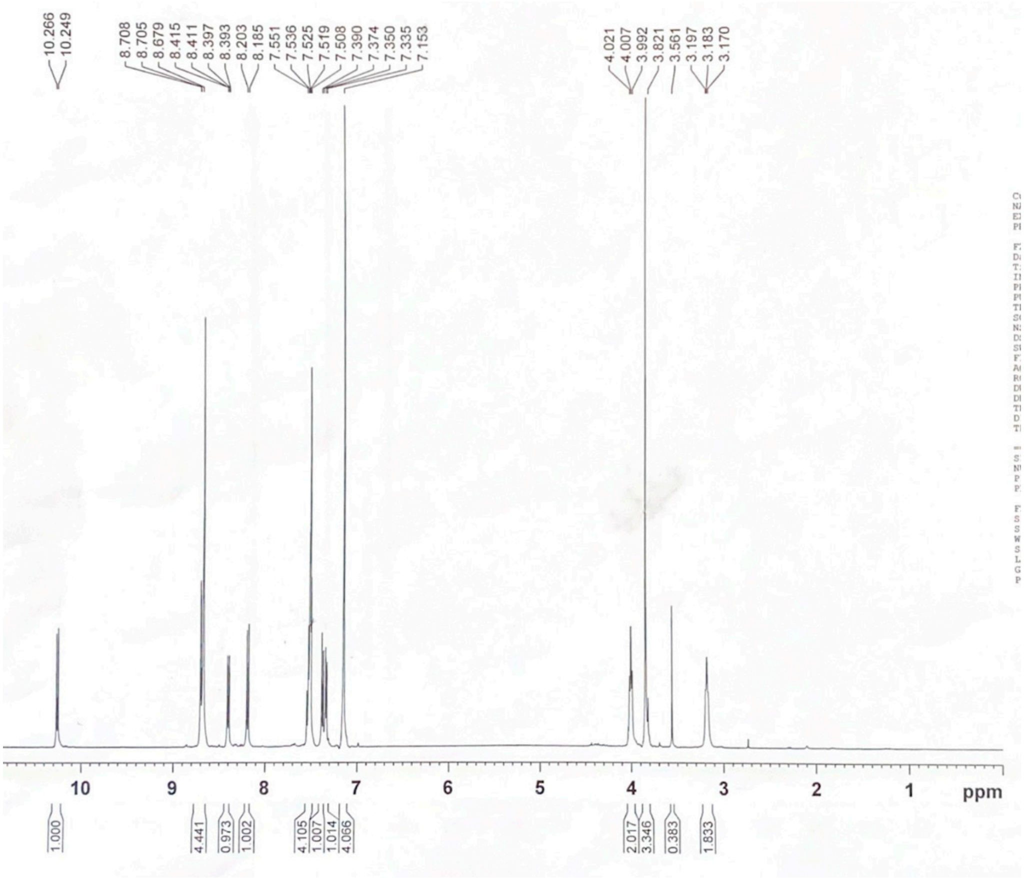

Figure S2b ^{13}C NMR for compound **3b**

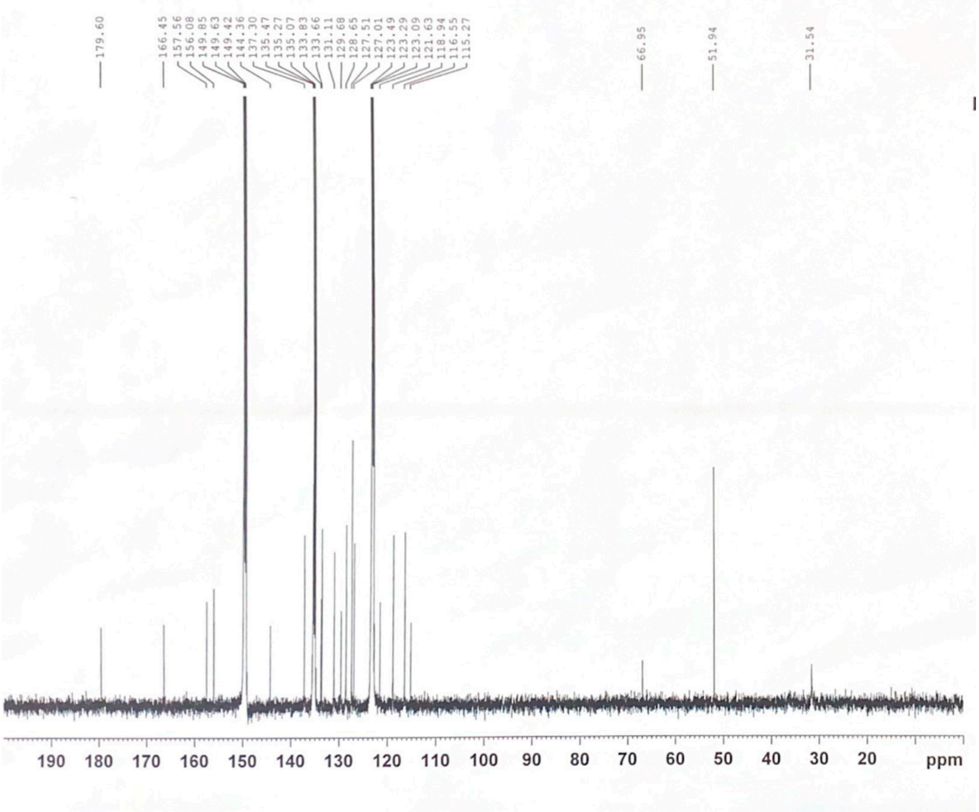

Figure S2c HRMS for compound **3b**

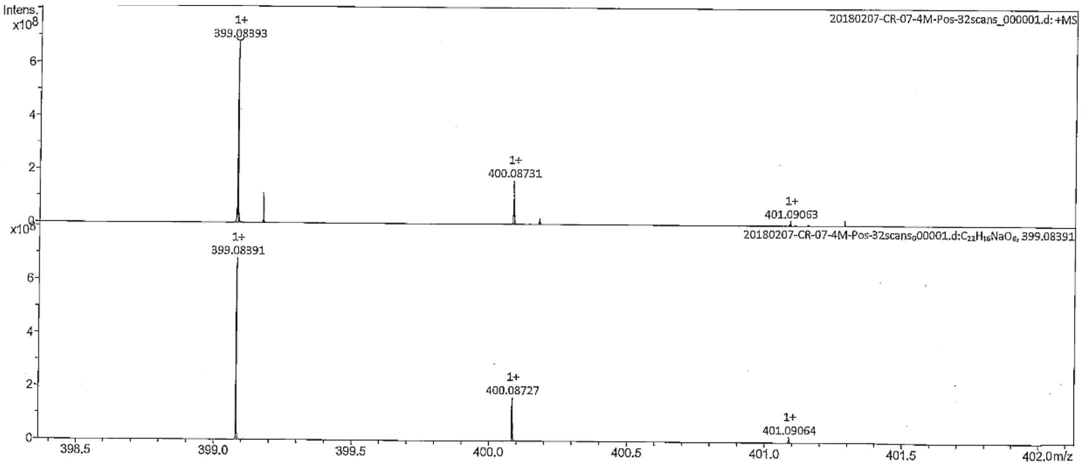

Figure S3a ^{1}H NMR for compound **3c**

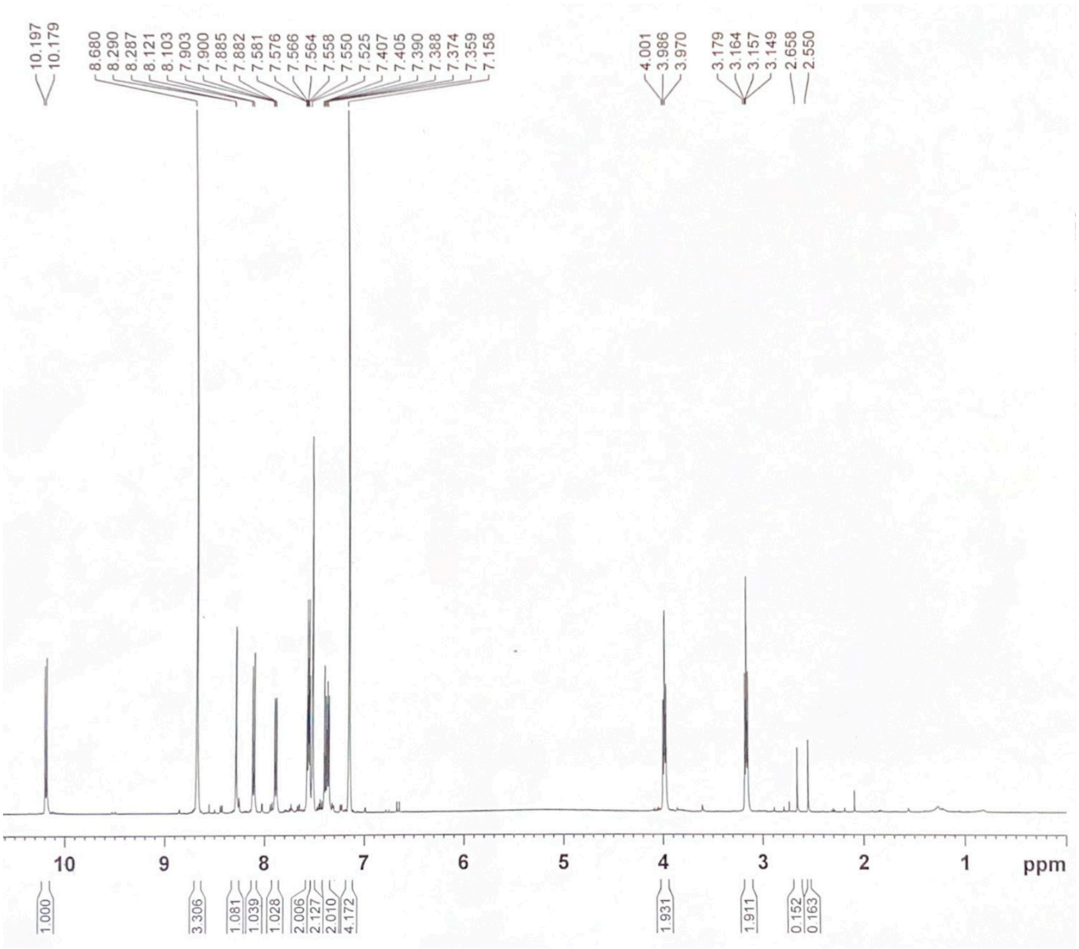

Figure S3b ^{13}C NMR for compound **3c**

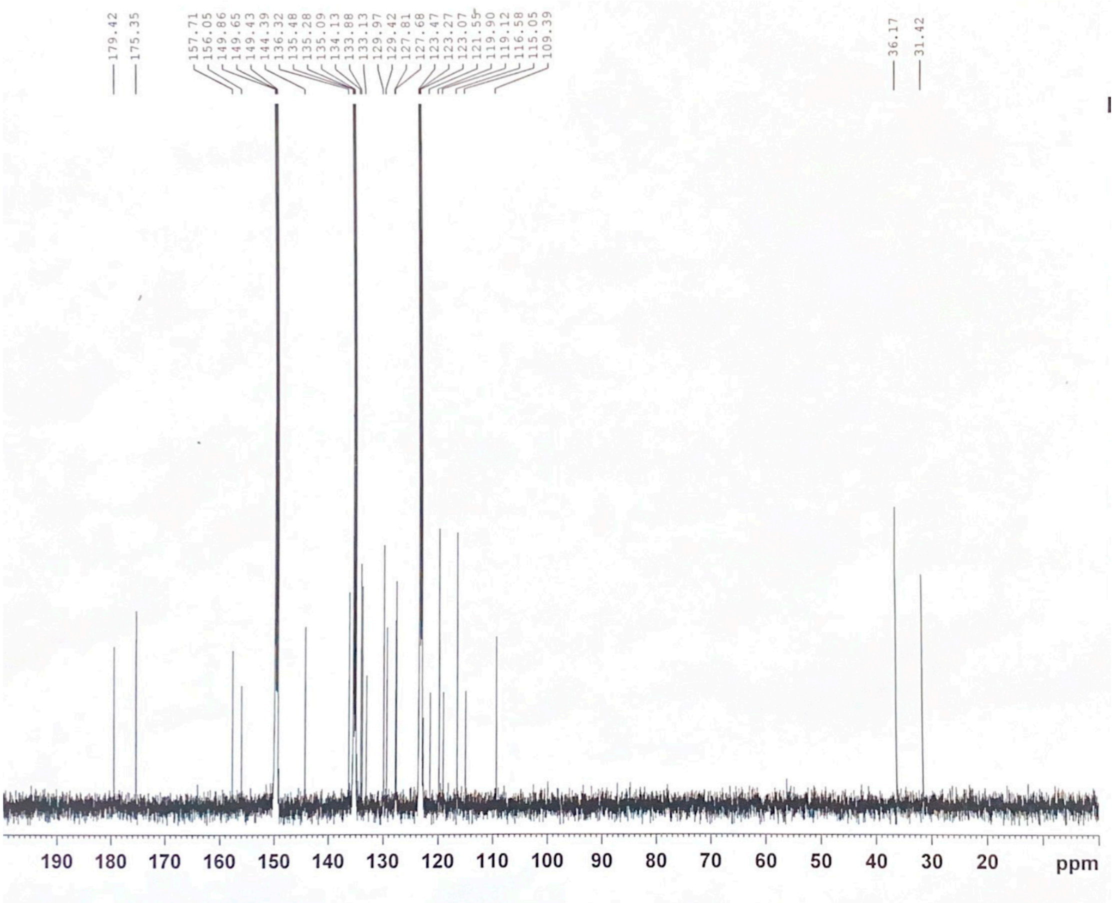

Figure S3c HRMS for compound **3c**

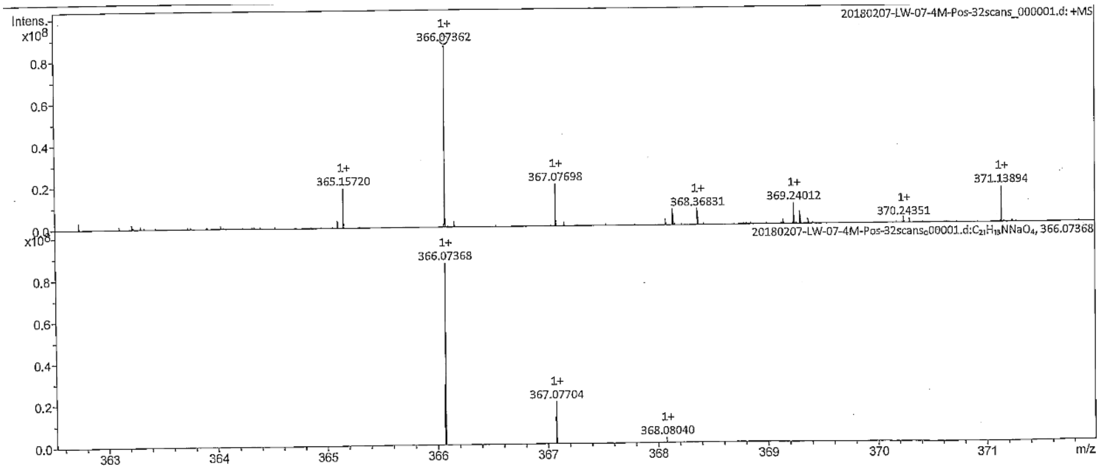

Supplement: Supplementary file 1 [file molecules-27-01970-s001.zip › molecules-1628426-supplementary.pdf]
